# Bladder cancer and the Notch pathway

**DOI:** 10.18632/oncotarget.3183

**Published:** 2015-01-26

**Authors:** Pablo J. Fernandez-Marcos, Manuel Serrano, Antonio Maraver

**Affiliations:** Institute de Recherche en Cancérologie de Montpellier (IRCM), Montpellier, France

The implication of the Notch pathway in cancer has been known since it was found hyperactivated in acute T-cell lymphoblastic leukemias (T-ALL) one decade ago. During this time, the link between the Notch pathway and cancer has been extended to many types of malignancies. A unique feature of the pathway is the fact that it can be oncogenic or tumor suppressive depending on the tumor type. For example, NOTCH receptors present gain-of-function mutations that make the receptor constitutively active in T-ALL, B-cell chronic lymphocytic leukemia, or lung adenocarcinoma. In contrast, NOTCH receptors undergo loss-of-function mutations in myeloid leukemias and in squamous cell carcinomas (SCCs) from esophagus, skin, lung or head and neck [1].

Two recent reports, from the group of Dr. Klinakis [2] and from us [3], have addressed the role of the Notch pathway in urinary bladder cancer demonstrating that it plays a relevant tumor suppressive role. The Klinakis group performed an extensive mutational analysis of up to nine components of the Notch pathway and copy number variations of the *NOTCH1* gene in a cohort of human bladder cancers [2]. Remarkably, they found that up to 60% of bladder cancers have loss-of-function alterations in components of the Notch pathway (43% overall mutation incidence, being more than half concentrated in *NOTCH1* and *NOTCH2*) and *NOTCH1* gene copy losses. In support of this, we also showed that *NOTCH1* and *NOTCH2* mutations previously found in bladder cancers are functionally defective [3]. Of further relevance, both studies found a correlation between low activity of the Notch pathway, higher cancer aggressiveness and shorter patient survival [2,3]. To support these findings, the two groups used mouse genetics to demonstrate that inactivation of the canonical Notch pathway at different levels promotes the development of bladder cancer in mice [2,3]. As it was the case in human cancers, Notch-deficient murine bladder cancers were also highly infiltrating [2,3].

The two studies performed mechanistic analyses that shed light on different aspects of the biology of Notch in bladder cancer. We focused on the aggressive behavior of the cancers with loss of Notch function. By manipulating the Notch pathway in cultured cells, we found that inhibition of the Notch pathway results in the upregulation of key mediators and effectors of the epithelial-mesenchymal-transition (EMT), including *SNAIL*, *SLUG*, *ZEB2* and *VIMENTIN*, and the concomitant downregulation of the epithelial marker *E-CADHERIN* [3]. The transcriptional repressor HES1 is one of the critical transcriptional targets of the Notch pathway, and we showed that reduced HES1 activity upon Notch inhibition is responsible for the de-repression of the EMT program in bladder cancer cells [3]. Therefore, we conclude that Notch represses EMT in bladder, where it acts as a tumor suppressive pathway. In this regard, it is interesting to note that a large body of evidence indicates that Notch induces EMT in those tissues where Notch is oncogenic. It appears that Notch acts in opposite directions in different tissues: it represses EMT in tissues where Notch is tumor suppressive; and it activates EMT in tissues where it is oncogenic. We will come back to this issue at the end of this commentary.

A sizeable proportion of human bladder cancers have gain-of-function mutations in FGFR3, HRAS, KRAS or PIK3CA genes. These mutations potently activate the canonical MAPK cascade and, accordingly, are associated to high levels of phospho-ERK (pERK1/2). The Notch signaling pathway is not generally considered a potent activator of the MAPK cascade. Unexpectedly, Klinakis' group made the intriguing observation that Notch-deficient bladder cancers (with no other concomitant mutations in FGFR3 or RAS) present high levels of pERK1/2, even higher than those in FGFR3/RAS-mutant bladder cancers [2]. Mechanistic analyses in culture cells led the authors to conclude that the Notch pathway transcriptionally activates several members of the dual-specificity phosphatases (DUSPs), including DUSP1, by direct binding of the activated NOTCH receptors to their promoter regions [2]. The DUSP phosphatases are responsible for the dephosphorylation of pERK1/2 and, therefore, when the Notch pathway is not functional DUSP expression decreases and there is an increase of pERK1/2 in bladder cancer cells [2]. Of note, previous work from others and us in lung adenocarcinoma, where Notch is oncogenic, has shown that the Notch pathway, through HES1, represses the DUSP1 promoter [4,5]. Again, as in the case of EMT, it appears that Notch acts in opposite directions depending on the tissues: it activates DUSPs in tissues where it is tumor suppressive; and it represses DUSPs in tissues where it is oncogenic.

Based on the above-described sets of mechanistic studies, it is tempting to speculate that the dual actions of the Notch pathway, oncogenic or tumor suppressive, reflect opposite actions on the same transcriptional targets. This situation is not new to researchers working on Notch in *Drosophila*, where it is well established that the Notch pathway can act positively and negatively on the same genes. Specifically, Notch-regulated genes often have binding sites for NOTCH/RBPJ (positive) and for the Notch target HES1 (negative), and this is thought to allow transient transcriptional effects [6]. In this context, alterations in the activation/repression equilibrium in different cell types may dramatically change the outcome of Notch activity. For example, this may well be the case of *DUSP1*: in those settings where the Notch pathway is tumor suppressive (as in bladder), the activation promoted by NOTCH1 is dominant; in contrast, in tissues where Notch is oncogenic (as in lung adenocarcinoma), the repressive action of HES1 is the dominant one. Conceivably, understanding the epigenetic status of critical Notch target genes could eventually explain the function, oncogenic or tumor suppressive, of the Notch pathway in cancer.

In summary, a new tumor suppressive role has been found for the Notch pathway in the urinary bladder, and this has yielded important mechanistic insights about bladder cancer and the biology of the Notch pathway.
